# Keratinocyte-derived CXCL-CXCR2 ligand-receptor axis by MED1 orchestrates neutrophil phenotypes during psoriasis

**DOI:** 10.3389/fimmu.2026.1935554

**Published:** 2026-09-30

**Authors:** Yingxi Li, Junkai Huang, Guixin Wang, Jing Luo, Zhikai Cao, Lin Li, Qi Guo, Qianshu Yuan, Mingwei Li, Ziyu Zhang, Weibin Xing, Long Su, Lizhi Hu

**Affiliations:** 1 Health Science Center, Ningbo University, Ningbo, Zhejiang, China; 2 Immunology Department, Key Laboratory of Immune Microenvironment and Disease (Ministry of Education), Tianjin Medical University, Tianjin, China; 3 Tianjin Medical University Cancer Institute and Hospital, National Clinical Research Center for Cancer, Tianjin, China; 4 Department of Dermatology, Tianjin Academy of Traditional Chinese Medicine Affiliated Hospital, Tianjin, China; 5 Department of Ophthalmology, Second Hospital of Tianjin Medical University, Tianjin, China

**Keywords:** keratinocytes, ligand-receptor axis, neutrophil, psoriasis, ScRNA-seq

## Abstract

**Background:**

Psoriasis is a chronic inflammatory skin disease characterized by aberrant keratinocyte proliferation and dysregulated immune responses. Increasing evidence suggests that reciprocal communication between tissue-resident keratinocytes and infiltrating immune cells shapes the inflammatory microenvironment and governs disease progression. However, the molecular mechanisms underlying this immune-parenchymal crosstalk remain incompletely understood.

**Methods:**

Here, we generate epidermal-specific Med1 conditional knockout (cKO) mice using the Cre-loxP system and induce psoriasis model with imiquimod (IMQ). To characterize immune-parenchymal interactions, single-cell RNA sequencing (scRNA-seq) was performed on lesional skin from cKO and wild-type (WT) mice. The underlying mechanisms governing keratinocyte-neutrophil communication were further investigated using flow cytometry, CellChat analysis, dual-luciferase reporter assays, transwell migration assays, and qRT-PCR.

**Results:**

Epidermal deficiency of Med1 markedly alleviated IMQ-induced psoriasis, as evidenced by reduced epidermal thickness and diminished inflammatory cytokine production. The scRNA-seq result showed epithelial cells were reduced, accompanied with the significantly diminished neutrophil accumulation in cKO mice. Flow cytometry analysis also showed that CD45^+^Ly6G^+^ neutrophils were found to be the most pronounced decreased relative to the other immune cells in cKO mice after IMQ. To further explore the dynamic changes of neutrophils in psoriasis, three novel neutrophil subtypes were identified, among which Cluster Nb exhibited a prominent chemotactic phenotype and was substantially diminished in cKO mice. Cell-cell communication analysis further identified the keratinocyte-derived CXCL-CXCR2 ligand-receptor axis as a key mediator of neutrophil recruitment. Mechanistically, keratinocyte-derived Med1 transcriptionally bound to CXCL1/2/3, thereby promoting CXCR2-dependent neutrophil chemotaxis during psoriasis.

**Conclusions:**

Collectively, our findings identify previously unrecognized neutrophil subtypes and demonstrates that epithelial Med1 regulates neutrophil phenotypes through the keratinocyte-derived CXCL-CXCR2 ligand-receptor axis. These findings uncover a microenvironmental mechanism governing neutrophil reprogramming and provide new insights into immune-parenchymal ligand-receptor communication in psoriasis.

## Introduction

Psoriasis is a common, chronic and relapsing immune-related skin disease (1). On the molecular level, psoriasis is characterized by excessive keratinocyte proliferation and involves immune system dysfunction as a key contributor to its development (2). Some biologics targeting the IL-23/IL-17 pathway have demonstrated clinical efficacy. However, their broad use is limited by costs, side effects related to immunomodulation and disease recurrence, which is mostly caused by the reciprocal communication between tissue-resident keratinocytes and infiltrating immune cells shapes the inflammatory microenvironment and governs disease progression (3–5). However, the molecular mechanisms underlying this immune-parenchymal crosstalk remain incompletely understood.

Psoriasis cannot be only classified as a T-cell-dependent disease. It is now well-established that keratinocytes play a crucial role in initiating the early pathogenic events and are also important in sustaining the chronic phase of the disease (6). The early upstream events in psoriasis involve the activation of innate immunity responses, and keratinocytes serving as the “first-line responders” to the immune environment of psoriasis (7). Upon activation by trigger factors, keratinocytes become a source of innate immune mediators (3). The latter include cationic antimicrobial peptides, cytokines and chemokines active in the recruitment of leukocytes of innate immunity, such as dendritic cells, macrophages, mast cells and neutrophils (8).

As the primary cells in innate immunity, neutrophils play a crucial role in causing immune microenvironment dysregulation and promoting the progression of psoriasis (9). In healthy states, neutrophils are released from the bone marrow into the circulation to surveil skin homeostasis, whereas in psoriasis, neutrophils are attracted into lesional skin and activated to release inflammatory mediators and neutrophil extracellular traps. These effector molecules and structures may activate resident cells such as keratinocytes, vascular endothelial cells and other immune cells to cause hyper inflammations (10). Moreover, multiple clinical studies have demonstrated that neutrophil-targeted therapies can exert their effects on psoriasis treatment (11–13). Above all, neutrophils play a significant role in the progression of psoriasis. However, it still remains a complicated question of the crosstalk between keratinocytes and neutrophils.

Serving as a molecular transcriptional bridge, Mediator 1 (Med1), plays an important role in mediating transcriptional signals from DNA binding transcription factors to RNA polymerase II (14). In our previous study, Med1 is high expressed in keratinocytes. The deficiency of Med1 could regulate the epidermal proliferation, disrupt the hair cycle and affect melanoma progression (15, 16). In this study, with Med1 conditional knockout mice, we found that the deficiency of Med1 in the epidermis alleviated IMQ-induced psoriasis by inhibiting keratinocytes proliferation and impairing neutrophils accumulation. Moreover, single-cell RNA sequencing (scRNA-seq) was performed to explore the crosstalk between epithelial cells and multiple immune cells in cKO and WT mice after IMQ. Cell-cell communication analysis further identified the CXCL-CXCR ligand-receptor axis between keratinocytes and neutrophils. To further explore the dynamic changes of neutrophils, three novel neutrophil subtypes have been identified and demonstrated that neutrophil Cluster Nb has potential roles on neutrophil chemotaxis in the development of psoriasis. Finally, we elucidated the regulatory mechanism underlying neutrophil chemotaxis of Med1 deficiency on epidermis.

In summary, our findings provide a comprehensive understanding on the dynamic changes of neutrophils during the psoriasis at the single-cell level and identify the role of Med1 regulating the neutrophil phenotypes, thus providing important insights for optimizing targeted therapeutic strategies for psoriasis in the future.

## Materials and methods

### Experimental animals

The cKO mice and WT mice on the C57BL/6J background were kindly given from the University of California San Francisco (15). Sex- and age-matched C57BL/6J wildtype mice were obtained from SiPeiFu (Beijing, China) Biotechnology Co., LTD. The animal study protocol was reviewed and approved by the Institutional Animal Care and Use Committee (IACUC) of Tianjin Medical University. The study was conducted in accordance with the local legislation and institutional requirements.

To perform psoriasis model, mice were topically treated with IMQ (Med-shine Pharma, China) for consecutive 6 days. For ear skin tissues, 10 mg/day of IMQ was applied to both the front and back sides of each mouse ear. For dorsal skin tissues, each mouse received daily 62.5 mg IMQ.

### Cell culture

The cell lines present in this study were obtained from the American Type Culture Collection (ATCC). Human keratinocytes (HEK) were cultured with DermaLife basal medium, containing DermaLife K LifeFactors Kit (LM-0004, Lifeline, USA). To perform psoriatic model *in vitro*, M5 cocktail stimulation (10 ng/ml IL-1α, IL-22, OSM, IL-17A, TNF-α) were treated on HEK for 24h, all recombinant proteins were obtained from MedChemExpress (MCE, HY-P73145, HY-P7039, HY-P70465, HY-P70527, HY-P70426A). HL-60 neutrophils were cultured in IMDM medium (Gibco, 12440-053, USA) supplemented with 10% FBS (FS401-02, TransGen Biotech, China) and penicillin/streptomycin (G4003, Servicebio, China) at 37 °C, 5% CO_2_. HaCaT cells were cultured in DMEM medium containing 10% FBS and 1:100 penicillin/streptomycin (G4523, G8003, G4003, Servicebio, China).

### Single-cell isolation and RNA preparation

For each group, two *Med1^flox/flox^* mice and two *Med1^flox/flox^K14Cre* mice had imiquimod (IMQ) applied to their dorsal skin for three consecutive days to establish a psoriasis model. Using scissors and forceps sterilized by autoclaving, psoriasis-like lesions from the modeled area were excised; lesion edges were trimmed and subcutaneous fat was removed, then the samples were immediately placed in tissue preservation solution and sent to a biotech company for tissue dissociation. After dissociation into a single-cell suspension, cell counting and viability assessment were performed. If cell viability meet the requirements for single cell sequencing, the cell suspension was diluted to the appropriate volume for subsequent single-cell sequencing loading. Library preparation, quality control, and sequencing were then commissioned to Xunyin Company, China for data processing.

### Single-cell RNA sequencing

‘Seurat’ (v4.3.0) package was used to performed the quality control and downstream analyses for single-cell RNA sequencing. Strict and standard procedures were performed to obtain the high-quality cells as follow: 1. nFeature_RNA > 200; 2. nCounts_RNA > 500; 3. The percent of mitochondria genes < 10%. And potential doublets were removed by the ‘doubletFinder’ package. Subsequently, the ‘harmony’ package was used to reduce the batch effect among samples. The markers used for cell annotation were collected by the public research and database. Details in the analysis of tissue preferences can be found in published studies (17, 18).

### Pseudo-time and cell developmental analyses

The cell trajectory analysis was conducted by ‘SCP’ package (https://github.com/zhanghao-njmu/SCP). Concretely, the pseudo-time of cell trajectory was calculated by the slingshot algorithm. Also, the dynamic changes of lineage were analyzed by the ‘RunDynamicFeatures’ function in SCP package. The parameter of gene clusters was set as 6. In addition, the ‘Vector’ package was used to validate the cell developmental direction of neutrophils. The significant markers of each subgroup of neutrophils were identified by the ‘FindAllMarkers’ function. The markers with log2FC >0.5 and P < 0.01 were considered as significant.

### CellChat analysis

Cell-cell communication was inferred using CellChat with the mouse CellChatDB database. Communication probabilities between cell groups were calculated by the tri-mean method with population-size correction, and interactions involving <10 cells were filtered. Total interaction numbers between each cell-group pair were visualized as circle plots comparing cKO vs WT, where edge width reflects interaction number and vertex size reflects cell number. Communication strengths were compared statistically using merged CellChat objects.

### Flow cytometry

2 mg/ml Dispase II (D6430, Solarbio, China) was used for the separation of the ear epidermis from the dermis. To separate the epidermis, place the digested skin dermal side down on a sterile flat surface. Using fine forceps, gently peel the epidermis away from the dermis. The dermis was then digested with Collagenase IV (C5138, Merck, USA) and DNase I (11284932001, Merck, USA). When tissue is fully ground with a mortar pestle, it is suspended into single-cell with medium. Then cell suspension was pretreated with an Fc receptor-blocking antibody (anti-mouse CD16/32, 101301, Biolegend, 1:200, USA). Next, APC/Cy7-CD45 (157203, Biolegend, 1:200, USA), FITC-F4/80 (101205, Biolegend, 1:200, USA), Perp/Cy5.5-Ly6G (127615, Biolegend, 1:200, USA) were added at 4 °C for 1h for evaluating the infiltration of skin innate immune cells. Results were obtained using the BD LSR Fortessa (Becton, Dickinson and Company, USA). Gated strategy: samples were analyzed immediately after preparation, and during data analysis, we applied morphological gating based on forward scatter (FSC-A) and side scatter (SSC-A) parameters to exclude debris and obvious dead cell populations. We used BD LSR Fortessa to collect CD45^+^ immune cells from skin single-cell suspension. Then CD45^+^Ly6G^+^ cells were considered as neutrophils, CD45^+^F4/80^+^ cells were considered as macrophages, CD45^+^CD11c^+^ cells were considered as dendritic cells.

### Immunohistochemical (IHC) and Immunofluorescent (IF) staining

For clinical specimens, written informed consent was obtained from all participants, and the study protocol was approved by the hospital’s ethics committee (LLKY2022-39, Tianjin Academy of Traditional Chinese Medicine Affiliated hospital, Tianjin, China). The study was conducted in accordance with the local legislation and institutional requirements.

IHC was performed as previously described (19) and incubated with anti-Med1 antibody. For histological examination, paraffin-embedded ear tissue was cut into 5 μm sections to perform H&E staining (G1120, Solarbio, China) and immunohistochemistry staining according to previous protocols (20). For immunofluorescent staining, slides were incubated with anti-ki67 antibody (GB121141-100, Servicebio, 1:200, China), anti-MPO antibody (22225-1-AP, Proteintech, 1:200, China) overnight at 4 °C. After that, Multi-rAb CoraLite^®^ Plus 488-Goat Anti-Rabbit/Mouse Recombinant Secondary Antibody (RGAR002, RGAM002, Proteintech, 1:1000, China) were used. The samples were then incubated with secondary antibodies for 1 hour at room temperature, followed by nuclear staining with DAPI (P36971, ThermoFisher, USA).

### Data preprocessing

The GSE78097, GSE53552, GSE178228 and GSE117239 datasets were sourced from the GEO database.

### CIBERSORT

CIBERSORT was used to estimate immune cell fractions from two pooled psoriasis cohorts after ComBat batch correction. Samples from GSE178228 and GSE117239 database (including 115 psoriasis samples) were divided into MED1-high and MED1-low groups by the median MED1 expression, and CIBERSORT neutrophil fractions were compared between the two groups by the Mann-Whitney U test, shown as box plots with individual sample points (n and P values indicated).

### Neutrophils transwell assay

200 μl dHL-60 neutrophils (2 × 10^5^) were suspended in serum-free culture medium (IMDM, 12440-053, Gibco, USA) and seeded into 24-well Transwell inserts (3.0 μm, 353096, Corning, USA). Conditioned media from keratinocytes cells (siMED1/siControl) after M5 stimulation and IMDM medium (1:1) were added to the remaining receiver wells. After 4 hrs (dHL-60 neutrophils and human keratinocytes HEK cells) or 12 hrs (dHL-60 neutrophils and mouse keratinocytes HaCaT cells), the migrated neutrophils were counted under the microscope.

### ELISA

Human CXCL1 (#EK196, Liankebio, China), human CXCL2 (#EK1264, Liankebio, China) and human CXCL3 (#EK1265, Liankebio, China) in the supernatants were measured by the ELISA kits according to the manufacturer’s instructions.

### Luciferase reporter assay

The CXCL1/2/3 promoter were cloned into the pGL3-basic vector (Promega, USA) upstream of the Firefly luciferase reporter gene. For dual-luciferase reporter assays, HaCaT cells were seeded in 24-well plates and co-transfected with 0.5 μg of the respective promoter construct and 25 ng renilla luciferase reporter vector using Lipofectamine 3000 (Thermo Fisher Scientific, USA). After 48-hour incubation, cell lysates were analyzed using Dual Luciferase Reporter Assay Kit (Vazyme), with Firefly luciferase activity normalized to Renilla luciferase activity to account for transfection efficiency. All experiments were performed in triplicate.

### Real-time fluorescence quantitative PCR

Total RNA was isolated from the skin using TransZol Up reagent (ET111-01, TransGen Biotech, China). cDNA was synthesized with All-in-One FirstStrand cDNA Synthesis SuperMix for RT-PCR (AE341-02, TransGen Biotech, China). Then samples were performed with PerfectStart Green qPCR SuperMix on the 7500-Fast Real-Time PCR System (AQ602-24, TransGen Biotech, China). The relative mRNA levels of each sample were determined by the 2^-ΔΔCt^ method with the housekeeping gene-glyceraldehyde-3-phosphate dehydrogenase (GAPDH). The primer sequences for qRT-PCR are listed in **Table 1**.

**Table 1:** Primer sequences for qRT-PCR.

Primers	Forward	Reverse
Gapdh	CCACTCCTCCACCTTTGAC	ACCCTGTTGCTGTAGCCA
Il17a	GCTGACCCCTAAGAAACCCC	GAAGCAGTTTGGGACCCCTT
Il1β	CAAGCTTCCTTGTGCAAGTGTCT	TCAAAAGGTGGCATTTCACAGT
Il1α	CGAAGACTACAGTTCTGCCATT	GACGTTTCAGAGGTTCTCAGAG
Il6	CTGCAAGAGACTTCCATCCAG	AGTGGTATAGACAGGTCTGTTGG
Cxcl1	ACTGCACCCAAACCGAAGTC	TGGGGACACCTTTTAGCATCTT
Cxcl2	CCAACCACCAGGCTACAGG	GCGTCACACTCAAGCTCTG
Cxcl3	GAGCACCAACTGACAGGAGA	AGTCCTTTCCAGCTGTCCCTA
S100a8	AAATCACCATGCCCTCTACAAG	CCCACTTTTATCACCATCGCAA
S100a9	GCACAGTTGGCAACCTTTATG	TGATTGTCCTGGTTTGTGTCC
GAPDH	AGGGCTGCTTTTAACTCTGGT	CCCCACTTGATTTTGGAGGGA
CXCL1	TCCTGCATCCCCCATAGTTA	CTTCAGGAACAGCCACCAGT
CXCL2	GAAAGCTTGTCTCAACCCCG	TCTCCTAAGTGATGCTCAAACA
CXCL3	GAGCACCAACTGACAGGAGA	AGTCCTTTCCAGCTGTCCCTA

### Statistical analysis

The data were presented for at least three individual experiments and statistical analyses were performed using GraphPad Prism and SPSS. Statistical significance of differences was determined using unpaired two-tailed Student’s t-test (*P < 0.05; **P < 0.01; ***P < 0.001).

## Results

### Epidermal Med1 deficiency alleviated IMQ-induced psoriasis

We firstly detected the expression of MED1 in patients with psoriasis. Immunohistochemistry results showed in **Figure 1A** revealed that MED1 was mainly expressed on the epidermis and it was significantly overexpressed in patients with psoriatic skin tissues compared with healthy donors. These results were also confirmed by the Gene Expression Omnibus (GEO) databases, which including 27 psoriasis samples and 6 control samples (**Figure 1B**). To further explore the function of Med1 in the epidermis of psoriasis, we construct the Med1 conditional knockout mice that Med1 was deleted on epidermis. The gene-targeting strategies were shown in **Figure 1C**. *Med1 ^flox/flox^* mice were inter-crossed with transgenic mice expressing K14 Cre recombinase under the control of K14 promoter. Cre positive (+/-) and flox positive (+/+) mice were selected as the experimental group (named as cKO). Cre negative (-/-) and flox positive (+/+) mice were used as the control group (named as WT). In cKO mice, Med1 were successfully deleted in the epidermis after confirmed by the protein and mRNA level using Western blot and PCR according to our previous work (15).

**Figure 1:**
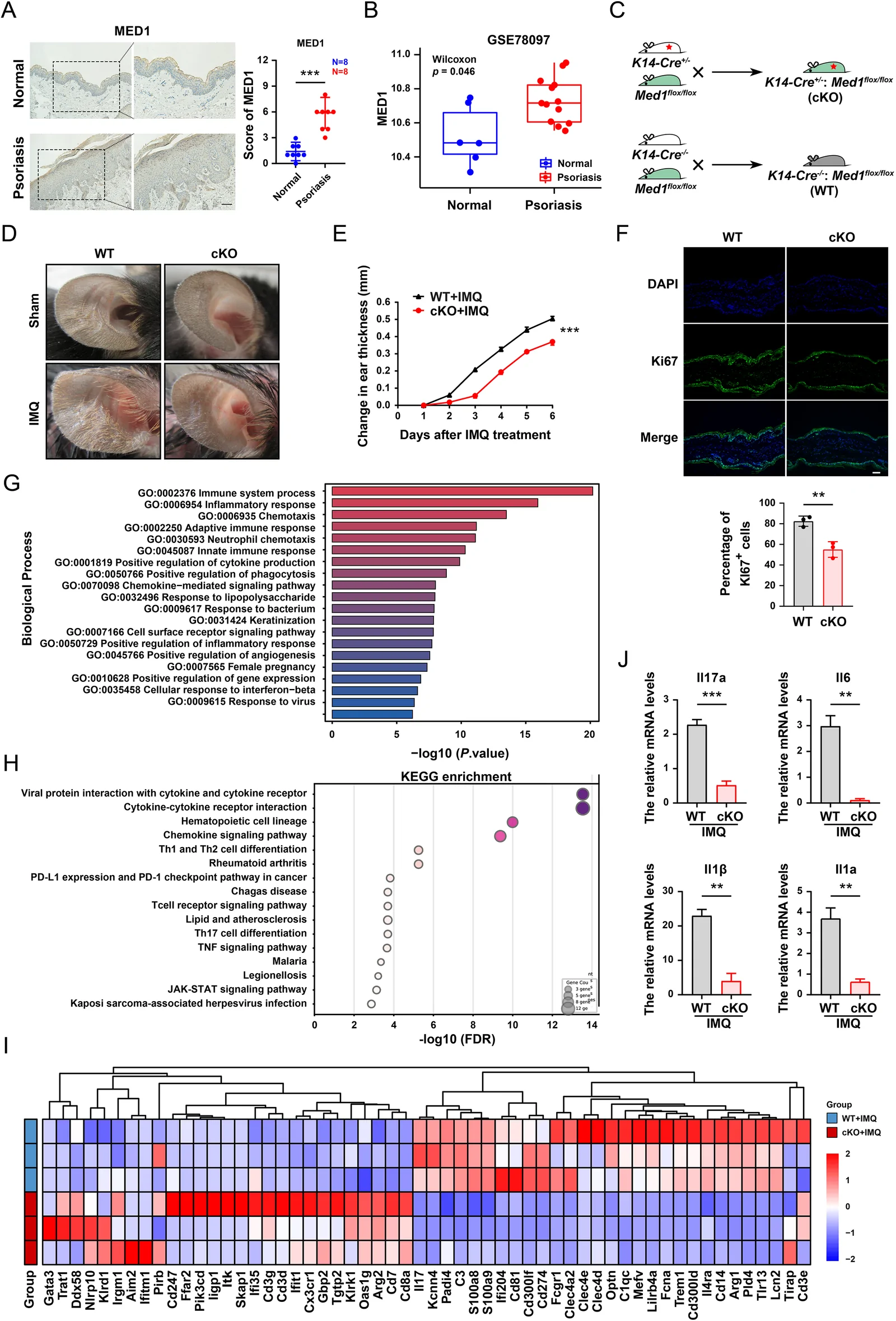
Med1 deficiency alleviated the severity of IMQ-induced psoriasis. **(A)** Representative images and statistical results of MED1 on immunohistochemistry staining in psoriasis and healthy donor’s skin tissues. n=8; **(B)** The expression levels of MED1 in psoriasis and normal tissues in GEO database; **(C)** The strategies of transgenic mouse model; **(D)** Representative images of mouse ear skin after treated with IMQ for 6 days. n=6; **(E)** Ear thicken of cKO and WT mice. n=6; **(F)** The expression of proliferate marker Ki67 in cKO and WT mice. Scale bar = 50 µm; **(G)** GO enrichment of biological process in IMQ-induced cKO vs WT mice; **(H)** KEGG analysis of IMQ-induced cKO vs WT mice; **(I)** The differential expressed genes of the immune system process in IMQ-induced cKO vs WT mice; **(J)** Expression of pro-inflammatory cytokines detected by RT-qPCR, respectively. *P < 0.05; **P < 0.01; ***P < 0.001.

In order to perform a psoriatic lesional skin in mice, we established a classic IMQ-induced psoriasis mouse model using cKO and WT mice. As is shown in **Figures 1D, E**, the severity of psoriasis was alleviated in cKO mice compared to WT mice, represented with less scaling and less epidermal thickness. When stained with Ki67 on psoriatic lesion in mice, we found Ki67 was low expressed in cKO than WT mice, suggesting that Med1 deficiency decreased the proliferate capacity on lesional psoriasis skin (**Figure 1F**).

To gain a comprehensive understanding of the transcriptional profile in IMQ-induced psoriasis cKO and WT mice, we conducted RNA sequencing in mice skin. In **Figure 1G**, Gene Ontology (GO) enrichment analysis showed several biological processes between IMQ-induced cKO and WT mice, including GO:0002376 (immune system process), GO:0006954 (inflammatory response), GO:0045087 (innate immune response), and GO: 0006934 (chemotaxis), which were significantly regulated by Med1 alterations in epidermis. KEGG analysis also showed that Cytokine-cytokine receptor interaction, Chemokine signaling pathway were enriched (**Figure 1H**). According to the immune system process, some pro-inflammatory cytokines and antimicrobial peptides were decreased in the skin of IMQ-treated cKO mice compared to WT mice (**Figure 1I**). From the RT-qPCR results, we found the expression of cytokines Il17a, Il6, Il1β and Il1α were decreased in IMQ-treated cKO mice (**Figure 1J**). Taken together, Med1 deficiency alleviated IMQ-induced psoriasis.

### Neutrophils accumulation was decreased in IMQ-induced cKO mice

In order to explore the crosstalk between epithelial cells and multiple immune cells in psoriasis, single-cell RNA sequencing (scRNA-seq) was performed in cKO and WT mice after IMQ. Firstly, strict and standard procedures of quality control of scRNA-seq were performed (**Supplementary Figure 1**). As shown in **Figures 2A, B**, 96786 high-quality single cells were clustered into 56 cell clusters, which were significantly distinguished. Based on marker gene expression, the t-distributed Stochastic Neighbor Embedding (tSNE) and Uniform Manifold Approximation and Projection (UMAP) analysis confirmed the separation of 11 cell types, including B cells, dendritic cells (DC), endothelial cells, mast cells, epithelial cells, macrophage cells, melanocyte cells, fibroblasts cells, neutrophils, natural killer cells (NK) and T cells (**Figures 2C, D**). The cell markers were sourced from the CellMarker database (**Figure 2E**). Further, as is presented by the cell preferences plot (**Figure 2F**) and proportion plot (**Figure 2G**), the distribution of cell clusters in these two groups showed that the percentages of epithelial cells were reduced in cKO mice, accompanied with the significantly diminished neutrophils. Cell counts between WT and cKO mice after IMQ from scRNA-seq data also showed that neutrophils were dramatically reduced in cKO mice (**Figure 2H**). To directly address the role of cell-cell communication, we performed CellChat analysis on the scRNA-seq data. Comparing with WT mice, we found that cell-cell communication between neutrophils and epithelial cells on cKO mice was markedly reduced (**Figure 2I**), indicating that Med1 deficiency impaired the crosstalk of neutrophil-epithelial cells.

**Figure 2:**
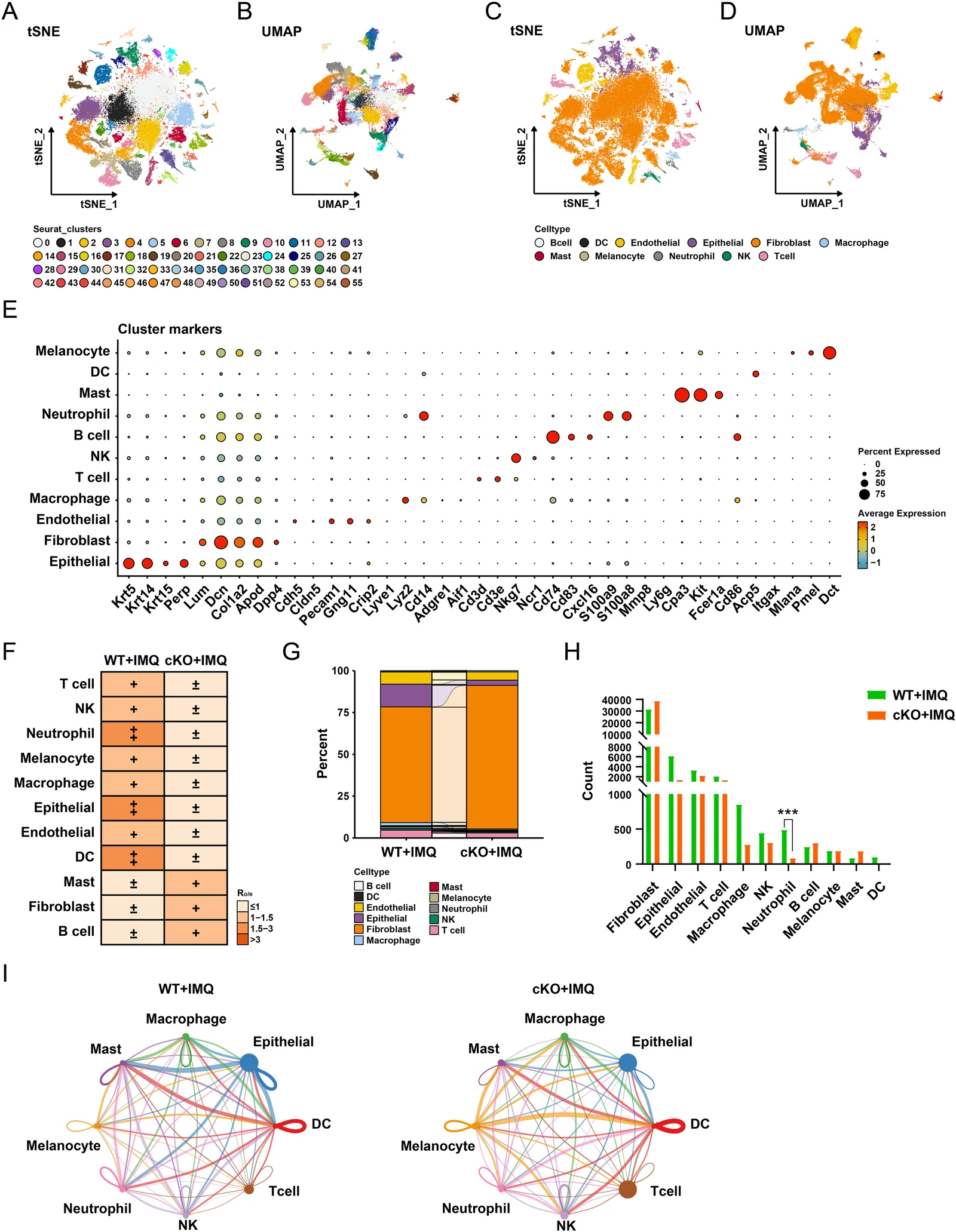
Different cell types in cKO and WT psoriatic skin mice were identified by scRNA-seq. **(A)** and **(B)** tSNE and UMAP scatter plots displayed 56 different cell clusters; **(C)** and **(D)** tSNE and UMAP scatter plots represented 11 different cell types; **(E)** Dot plots showed the expression levels of representative cell type-specific markers in the mouse skin used to note 11 different cell types. The dot size represented the percentage of cells expressing the selected gene in each population; **(F)** Tissue preferences of 11 different cell types between cKO and WT mice. Ro/e denotes the radio of observed to expected cell number. Ro/e ≤1, ±; 1<Ro/e<1.5, +; 1<Ro/e<3, ++; Ro/e>3, +++; **(G)** The proportion of corresponding cell types between cKO and WT mice; **(H)** Cell counts of corresponding cell types between cKO and WT mice; **(I)** CellChat analysis between cKO and WT mice. *P < 0.05; **P < 0.01; ***P < 0.001.

To further detect the immune cells accumulation, we performed Flow cytometry analysis to detect the infiltration of immune cells in psoriatic lesional mice induced by IMQ, such as CD45^+^Ly6G^+^ neutrophils, CD45^+^F4-80^+^ macrophages, CD45^+^CD11c^+^ dendritic cells and CD45^+^CD3e^+^ T cells. After exclude debris and dead cell populations, CD45^+^ immune cells, Ly6G^+^ neutrophils and F4-80^+^ macrophages were increased on psoriatic lesion in WT mice after induced by IMQ. However, when compared with WT mice, the percentage of CD45^+^Ly6G^+^ neutrophils were found to be the most pronounced decreased relative to the other immune cells in cKO mice after IMQ (**Figure 3A**), with CD45^+^CD3e^+^ T cells displaying slight changes (**Figure 3B**). Notably, these results demonstrated that the epidermal cKO mice significantly prevented CD45^+^Ly6G^+^ neutrophils accumulation on psoriatic lesion. To verify, we performed immunofluorescent staining to detect neutrophils infiltration. In **Figures 3C, D**, when induced by IMQ in WT mice, a large number of MPO^+^ neutrophils infiltrated on the epidermis and accumulated to form Munro microabscesses. However, fewer MPO^+^ neutrophils were observed in cKO mice, revealing that the epidermal cKO mice significantly prevented neutrophils infiltration. To validate the neutrophil infiltration in human psoriatic samples, we performed immune cell deconvolution using CIBERSORTx. The results demonstrated that the high-MED1 group exhibited significantly higher neutrophil infiltration compared to the low-MED1 group (**Figure 3E**). This finding is consistent with our mouse scRNA-seq data, which MED1 cKO mice showed reduced neutrophils accumulation.

**Figure 3:**
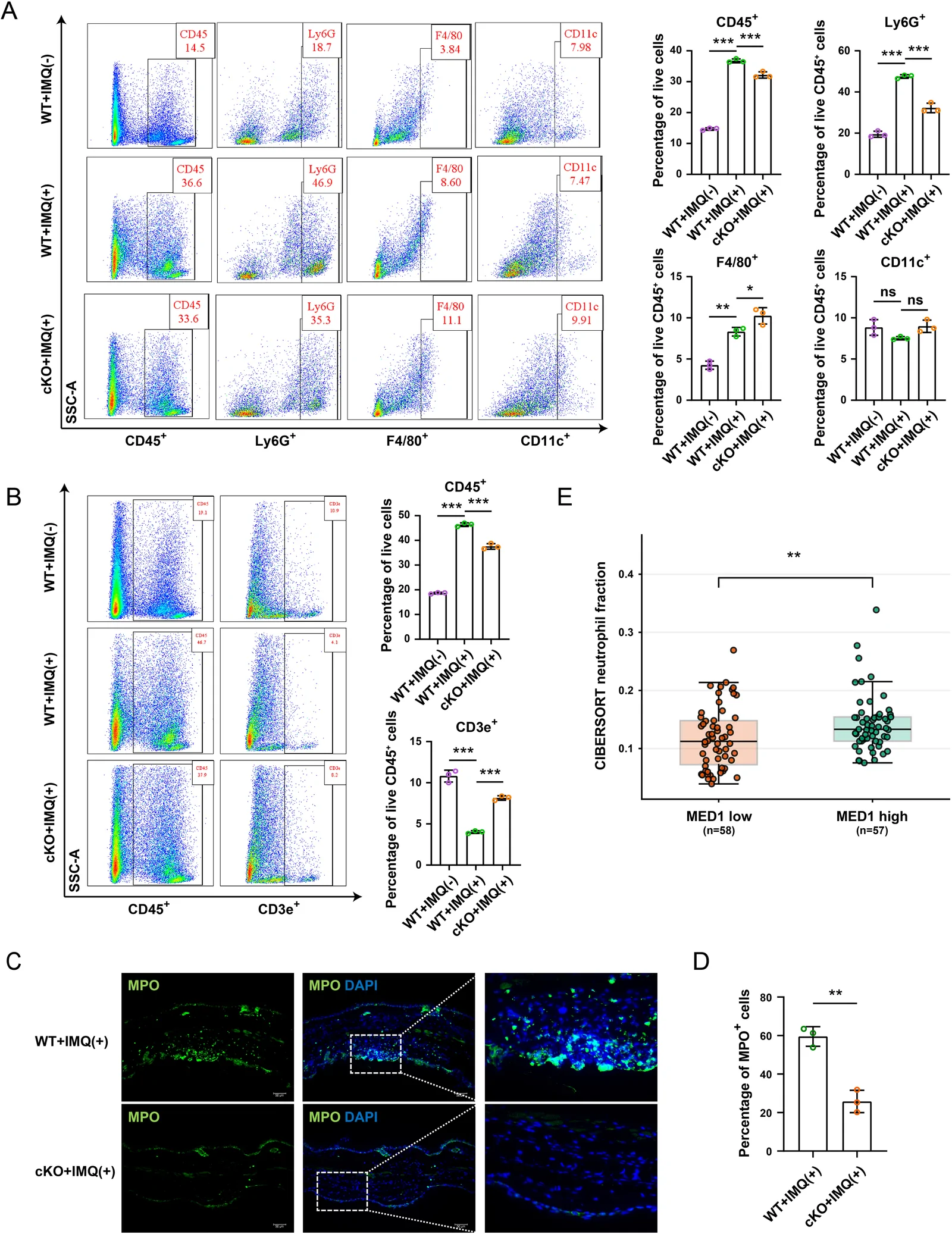
Neutrophil accumulation was decreased in IMQ-induced cKO mice by scRNA-seq. **(A)** and **(B)** Flow Cytometry analysis of innate immune cells **(A)** and T cells **(B)** on skin of cKO and WT mice after IMQ (n = 3) and its statistical results; **(C)** and **(D)** Immunofluorescent staining of MPO on cKO and WT mice ear and its statistical analyses. **(E)** Abundance of neutrophil infiltration between high- and low-MED1 expression groups in human samples from GEO database. *P < 0.05; **P < 0.01; ***P < 0.001.

### Med1 deficiency inhibited neutrophil accumulation on psoriatic lesion by decreased neutrophil chemotaxis in a CXCR2 dependent manner

Chemotaxis plays a significant role in the pathogenesis of psoriasis. It drives the recruitment of immune cells into psoriatic skin, amplifying inflammation and contributing to disease progression. Gene set enrichment analysis (GSEA) results showed that neutrophil chemotaxis was low enriched in the IMQ-induced cKO mice, suggesting that the inhibitory effect of epidermal Med1 deficiency on neutrophil accumulation maybe due to the diminished neutrophil chemotaxis (**Figure 4A**). The differential expressed genes according to the neutrophil chemotaxis between cKO and WT mice after IMQ-induced were showed in **Figure 4B**, including chemokines (Cxcl1, Cxcl2, Cxcl3, Cxcl9, Cxcl10), chemokine receptor Cxcr2 and neutrophil-derived inflammatory markers (S100a8, S100a9). The results from RT-qPCR also showed that the expression of Cxcl1, Cxcl2, Cxcl3, Cxcl9, S100a8 and S100a9 were significantly decreased in IMQ-treated cKO mice (**Figure 4C**). Our scRNA-seq data further demonstrated that Cxcl1, Cxcl2 and Cxcl3 were significantly down-regulated in cKO mice (**Figure 4D**). Moreover, we observed that Cxcr2, the receptor of Cxcl1/2/3, is predominantly expressed in the neutrophil (**Figure 4E**). Collectively, these findings suggest that the impaired neutrophil chemotaxis induced by Med1 deficiency may be associated with the CXCL1/2/3-CXCR2 axis.

**Figure 4:**
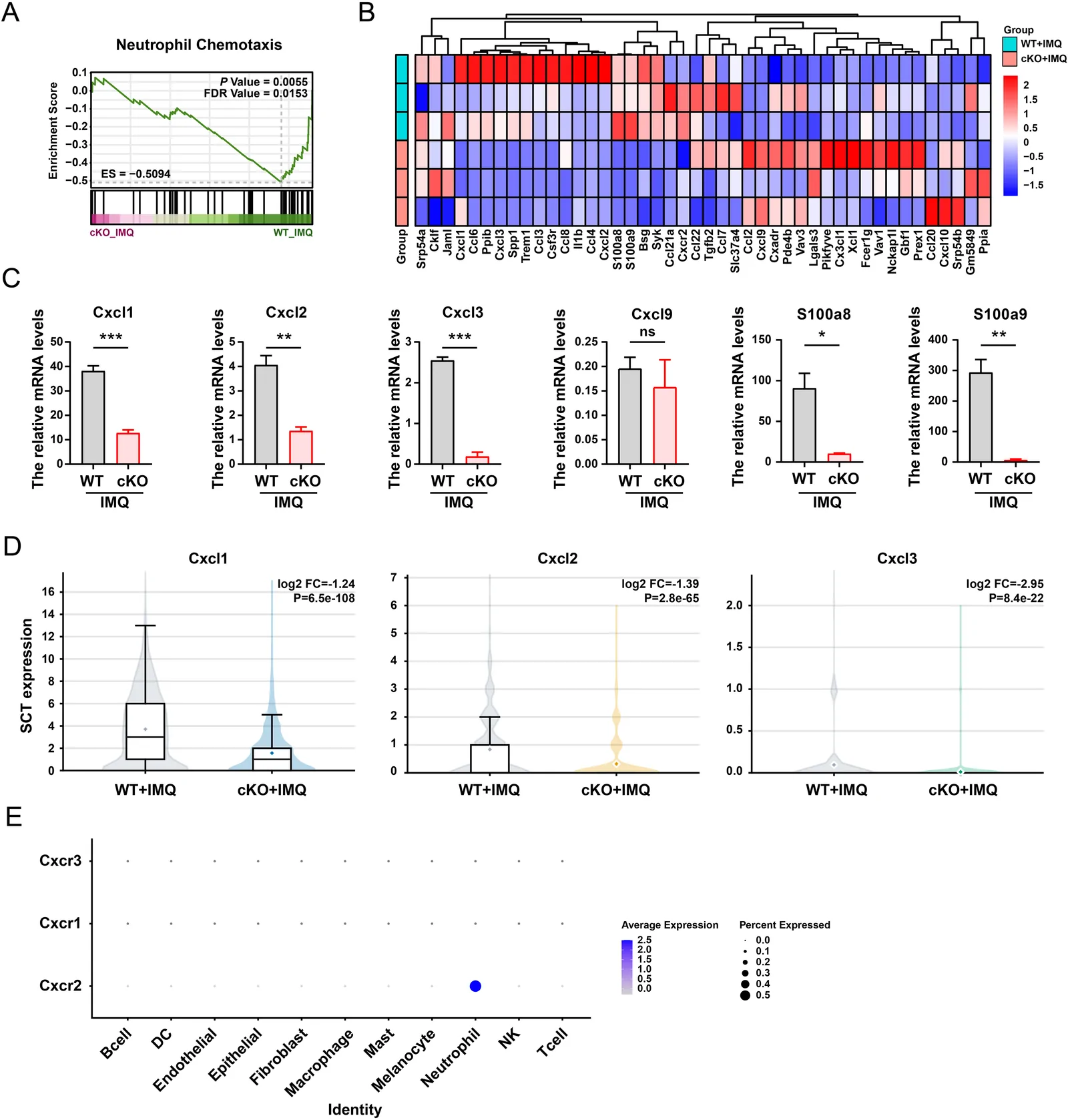
The diminished neutrophil accumulation on Med1 deficiency dues to neutrophil chemotaxis. **(A)** Gene set enrichment analysis of neutrophil chemotaxis in cKO and WT mice after IMQ induced; **(B)** Heat map representation of the upregulated and downregulated genes of neutrophil chemotaxis in cKO and WT mice after IMQ induced. Red and blue signal indicates high and low expression, respectively (n = 3); **(C, D)** Expression of chemokines detected by RT-qPCR **(C)** and scRNAseq **(D)**; **(E)** The expression levels of Cxcrs in the 11 different cell types by dot plots from scRNAseq. *P < 0.05; **P < 0.01; ***P < 0.001.

### The neutrophil subtypes Cluster Nb has potential role of neutrophil chemotaxis in IMQ-induced psoriasis

To further explore the dynamic changes of neutrophils in cKO and WT mice after IMQ, cell trajectory analysis was conducted. We identified three novel subtypes (Cluster of Na, Nb, Nc) of neutrophils. As shown in pseudo-time analysis, Cluster Na exhibited a potential evolution to Cluster Nc, while Cluster Nb might serve as an intermediate status (**Figure 5A**). These results were also verified by cell developmental analysis by ‘Vector’ package (**Figure 5B**). The characteristic marker genes of Cluster Na, Nb and Nc were computationally screened (**Figures 5C, D**). The marker gene Tmsb10 and Gpx3 were identified with the highest expression in Cluster Nb. We also found that Cluster Nb were higher enriched in WT mice group, however, the proportion of Cluster Nb were significantly diminished in cKO (**Figures 5E, F**). Further, in order to explore the functional characteristics of these three neutrophil subtypes, the dynamic gene expression analysis was performed (**Figure 5G**). Notably, Cluster Nb was significantly enriched in the neutrophil chemotaxis, leukocyte migration and chemokine-mediated signaling pathway. Moreover, GO analysis of Cluster Nb (**Figure 5H**) also verified that the molecular functions of Cluster Nb were related on chemotaxis, including leukocyte chemotaxis (GO:0030595), neutrophil chemotaxis (GO:0030593) and positive regulation of chemotaxis (GO:0050921). However, Cluster Nc was enriched in negative regulation of cell migration, negative regulation of cell motility, negative regulation of locomotion and focal adhesion (**Supplementary Figures 2A, B**). As we mentioned above, the impaired neutrophil chemotaxis induced by Med1 deficiency was associated with the CXCL1/2/3-CXCR2 axis, so we analyzed the expression of CXCR2 in neutrophil subtypes. We found that Cxcr2^+^ neutrophils of Cluster Nb were dramatically reduced in cKO mice compared with WT mice (**Figure 5I**). Taken together, these results indicated that Cluster Nb has potential role of neutrophil chemotaxis in IMQ-induced psoriasis.

**Figure 5:**
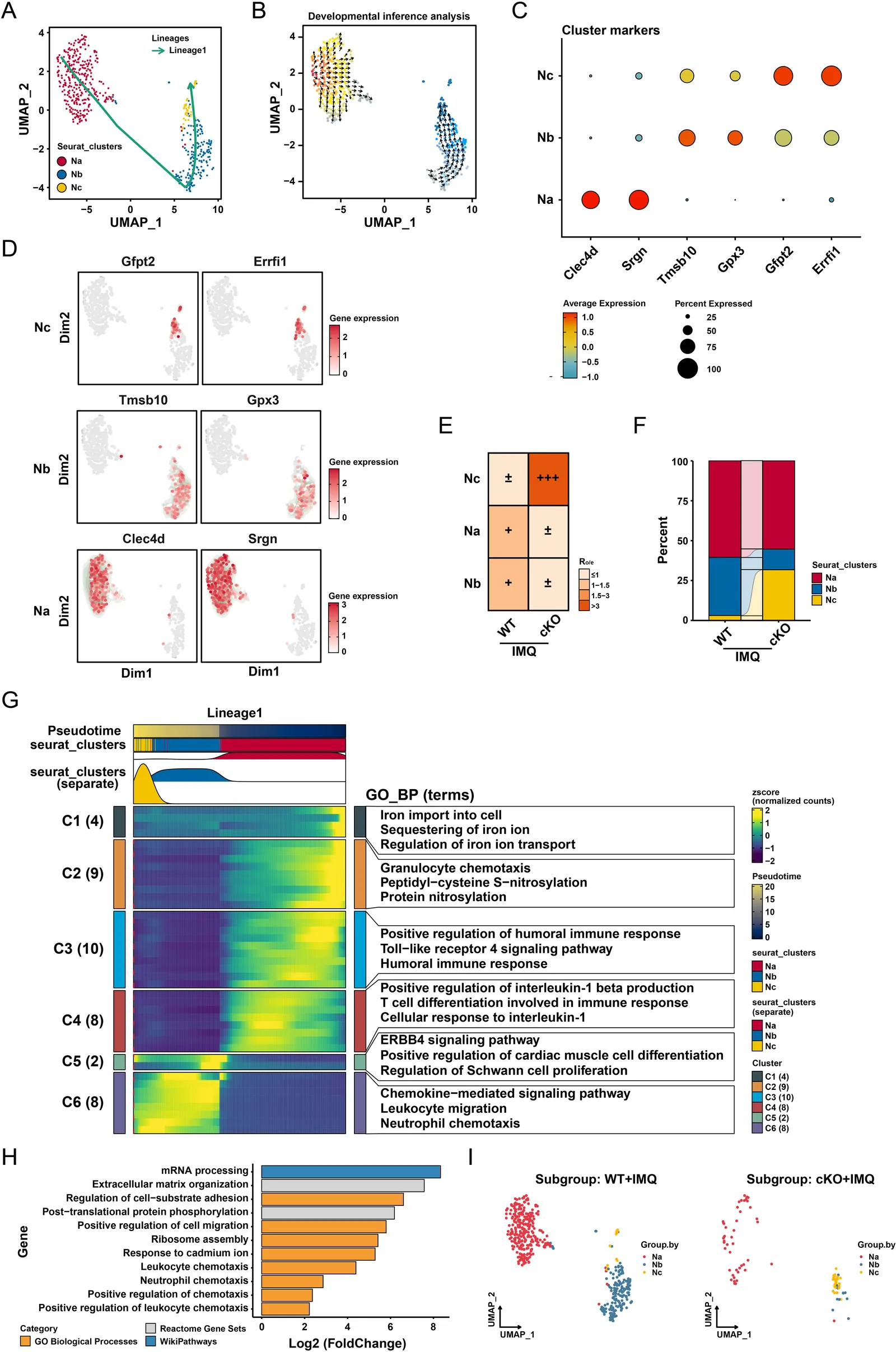
Cell trajectory and reprogramming of neutrophil subtypes in IMQ induced cKO and WT mice. **(A)** Cell trajectory of neutrophil was generated using the slingshot algorithm; **(B)** Cell developmental direction of neutrophil visualized by UMAP. Arrows represent the direction of cell development; **(C, D)** Dot plots **(C)** and feature plots **(D)** showing the expression of markers representative of the three neutrophil subgroups. **(E)** Tissue preferences of 3 different subgroups of neutrophils between cKO and WT mice after IMQ. Ro/e denotes the radio of observed to expected cell number. Ro/e ≤1, ±; 1<Ro/e<1.5, +; 1<Ro/e<3, ++; Ro/e>3, +++; **(F)** Alluvial diagram showing the cell proportion of 3 different subgroups of neutrophils; **(G)** The differential expressed genes (DEGs) with expression levels that changed the most over the pseudo-time trajectory were divided into six clusters based on their expression trend, and the representative processes of each cluster are shown. Color key from deep blue to yellow indicates relative expression levels of the DEGs from low to high. The numbers in parentheses after the cluster represent the trees of the gene; **(H)** GO analysis showing the pathways enriched in Cluster Nb; **(I)** UMAP scatter plots displayed Cxcr2^+^ neutrophils in three distinct subgroups.

### MED1 transcriptionally binds to CXCLs to promote neutrophil chemotaxis

To further explore the mechanism of neutrophil chemotaxis on epidermal Med1 deficiency, we mimic a psoriatic model with M5 cocktail stimulation (IL-1α, IL-22, OSM, IL-17A, TNF-α, 10 ng/ml) on human keratinocytes *in vitro*. After the stimulation, human keratinocytes were co-cultured with human neutrophils (**Figure 6A**). Interestingly, we found the number of neutrophils were significantly diminished when co-cultured with HaCaT (**Figure 6B**) or HEK cells (**Figure 6C**) in siMED1 group. These results indicate that Med1 deficiency on epidermis inhibited neutrophil accumulation on psoriatic lesion by decreased neutrophil chemotaxis. Moreover, we found chemokines CXCL1, CXCL2 and CXCL3 were decreased on MED1 down-regulated HaCaT cells after M5 stimulation (**Figure 6D**). The protein level of CXCL1, CXCL2 and CXCL3 detected by ELISA were also decreased on the supernatant of HaCaT cells (**Figure 6E**). In order to investigate how Med1 regulate the chemokines, dual-luciferase reporter assays were conducted. The promoter of CXCL1, CXCL2 and CXCL3 were cloned into the pGL3-basic vector with firefly luciferase reporter gene, respectively (**Figure 6F**). Dual-luciferase reporter assays demonstrated that MED1 deficiency significantly attenuated luciferase activity of CXCL1, CXCL2 and CXCL3 (**Figure 6G**). In addition, in order to verify the correlation between MED1 and CXCL1/2/3 in human samples, we found a positive correlation between the levels of MED1 and CXCL1, CXCL2 and CXCL3 from a GEO dataset (GSE78097 and GSE53552), involving 52 psoriatic lesion skin samples (**Figure 6H**). In conclusion, these results demonstrated that MED1 deficiency suppressed neutrophil chemotaxis by reducing its transcriptional binding to chemokines CXCL1/2/3.

**Figure 6:**
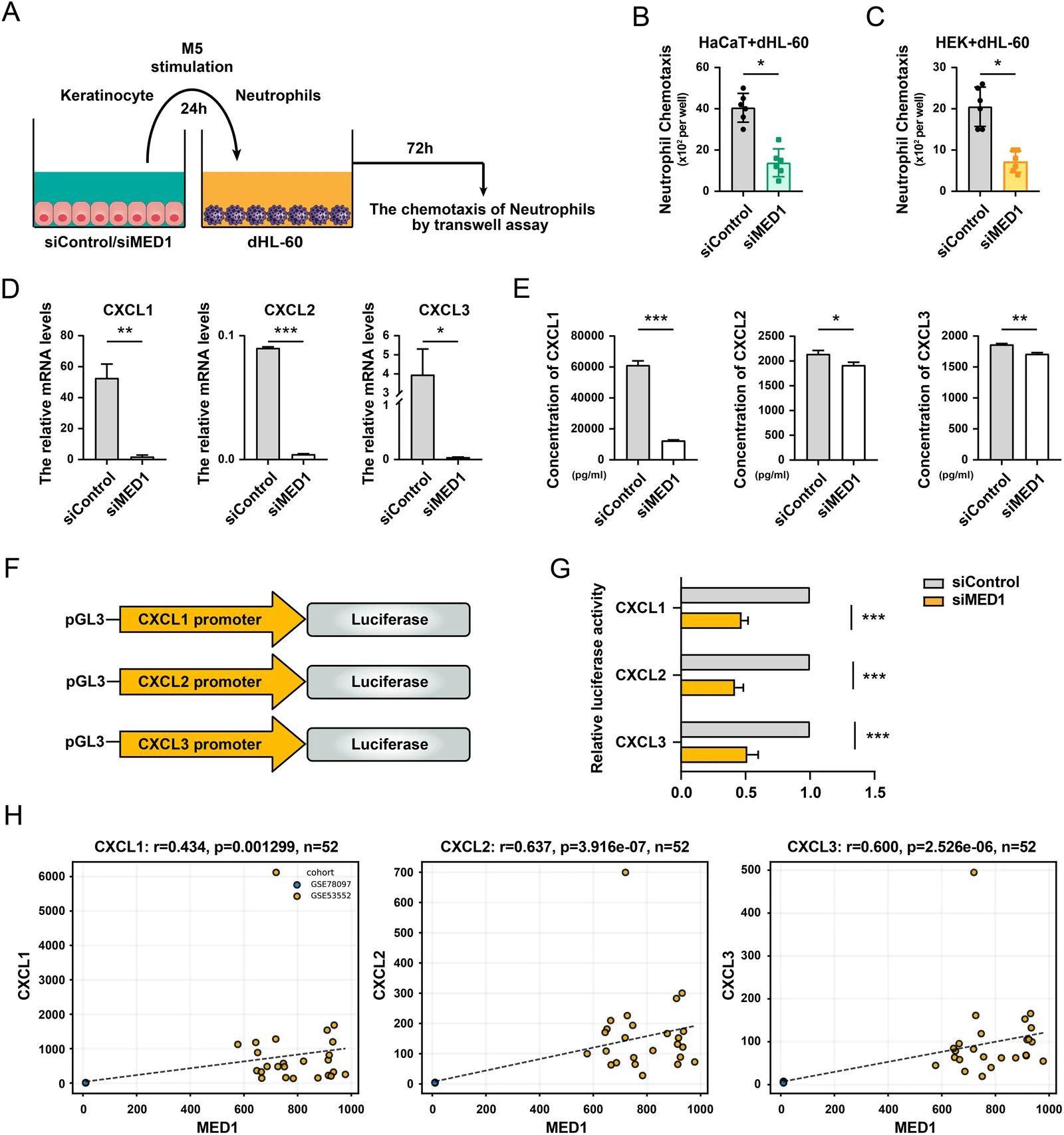
The transcriptional mechanisms of MED1 on neutrophil chemotaxis. **(A)** The *in vitro* co-culture model of human neutrophils (dHL-60) and human keratinocytes after M5 stimulation; **(B)** and **(C)** Transwell assay of neutrophils after co-cultured with human keratinocytes HaCaT **(B)** or HEK cells **(C)**. n=6; **(D)** qRT-PCR of CXCL1, CXCL2 and CXCL3 in the HaCaT cells with siControl and siMED1 group (n=3); **(E)** ELISA of CXCL1, CXCL2 and CXCL3 concentrations on the supernatant of HaCaT cells with siControl and siMED1 group (n=3); **(F)** The schematic diagram of CXCL1/2/3 promoter region into pGL3-basic plasmid; **(G)** The luciferase activity of CXCL1/2/3 on siControl and siMED1 group (n=3). **(H)** The correlation between MED1 and CXCL1/2/3 was calculated using Pearson correlation analysis from a GEO dataset. *P < 0.05; **P < 0.01; ***P < 0.001.

## Discussion

Psoriasis is a prevalent, chronic, and recurrent immune-mediated skin disorder that is not exclusively categorized as a T-cell-dependent disease (21). Keratinocytes, the predominant cells in the epidermis, play a crucial role in the pathogenesis of psoriasis. They not only serve as physical barriers but also actively participate in immune responses. In psoriatic conditions, keratinocytes exhibit abnormal proliferation and differentiation, contributing to the characteristic thickening and scaling of the skin. Moreover, these cells secrete various inflammatory mediators, including cytokines and chemokines, which further amplify the inflammatory cascade by recruiting immune cells to the skin. This dynamic interaction between keratinocytes and immune cells establishes a self-perpetuating inflammatory loop, leading to the persistence and exacerbation of psoriatic lesions (22, 23). In our study, after treatment with IMQ in WT and cKO mice, we found Med1 deficiency on keratinocytes could alleviate IMQ-induced psoriasis by decreasing the keratinocytes proliferation. Moreover, Med1 deficiency could reduce multiple inflammatory cytokines, such as Il17a, Il6, Il1β and Il1α (**Figure 1**).

Psoriasis is a chronic inflammatory skin disorder, featuring hyperproliferation of keratinocytes, increased angiogenesis and infiltration of various innate and adaptive immune cells (24). A recent single-cell study based on the spatial distribution of immune cells in the epidermis and dermis revealed that the immune microenvironment formed in the epidermis aligns more closely and significantly with the inflammatory responses observed during psoriasis, compared to the dermis (25). This suggests that the epidermis plays a dominant role in the development of psoriasis. In order to further explore the role of epidermal Med1 deficiency on the multiple immune cells in psoriasis, we performed scRNA-seq in cKO and WT mice after IMQ. There are 11 cell types, including epithelial cells, B cells, DC, endothelial cells, fibroblasts cells, macrophage cells, mast cells, melanocyte cells, neutrophils, NK and T cells were well separated. Importantly, both the cell percentage and cell number of neutrophils were significantly diminished in cKO mice (**Figure 2**). Flow cytometry analysis further showed that Med1 deficiency in epidermis could inhibit neutrophil accumulation on psoriatic lesion, represented by significantly decreased CD45^+^Ly6G^+^ neutrophils in cKO mice. Meanwhile, CD45^+^Ly6G^+^ neutrophils exhibited the most pronounced changes relative to the other inmmune cells (**Figure 3**). These results indicated that Med1 is a key regulator of the crosstalk between keratinocytes and neutrophils in psoriasis. Neutrophils accumulation in psoriasis was significantly decreased in Med1 deficiency on epidermis. Although the function of neutrophils in psoriasis is important, however, there is no change of Med1 expression in neutrophils in cKO mice. According to our cKO mice is an epidermal specific Med1 knockout mice, the expression of Med1 is only decreased in keratinocytes on epidermis and there is no change in neutrophils. These findings further indicate that the neutrophil accumulation induced by Med1 is primarily driven by keratinocytes in the epidermis, rather than by a direct effect of Med1 on the neutrophils. According to the macrophage infiltration, we have performed bioinformatic analyses on macrophage subpopulations from the WT and cKO groups. We compared the transcriptomic differences between the two groups and identified significantly upregulated differentially expressed genes in the cKO group. Subsequent functional enrichment analysis of these upregulated genes revealed significant enrichment in pathways related to cell-substrate adhesion, extracellular matrix organization, negative regulation of cell motility, however, there is no immune-related pathways (**Supplementary Figure 3**). So, we didn’t further investigate this in the ongoing studies.

Neutrophil recruitment is a hallmark of psoriatic inflammation and is largely orchestrated by immune-parenchymal communication within the cutaneous microenvironment. In the present study, epidermal Med1 deficiency markedly reduced the expression of multiple neutrophil-associated chemokines, including Cxcl1, Cxcl2, and Cxcl3, together with neutrophil activation markers S100a8 and S100a9 at inflammatory lesions. GSEA results also showed that neutrophil chemotaxis was low enriched in the IMQ-induced cKO mice. Notably, Cxcr2, the principal receptor for CXCL1/2/3 (26), was predominantly expressed by neutrophils, supporting the existence of a keratinocyte-derived CXCL1/2/3-CXCR2 ligand-receptor axis that mediates epithelial-neutrophil communication (**Figure 4**). These findings suggest that the impaired neutrophil chemotaxis induced by MED1 deficiency may be associated with the CXCL1/2/3-CXCR2 axis. Furthermore, single-cell trajectory analysis identified three previously unrecognized neutrophil subtypes (Clusters Na, Nb, and Nc). Among them, Cluster Nb was preferentially enriched in WT mice but markedly diminished in cKO mice, indicating that this population represents a chemotactic neutrophil phenotype maintained by keratinocyte-derived CXCL-CXCR2 signaling. Collectively, these results suggest that epithelial Med1 not only regulates neutrophil recruitment but also shapes neutrophil phenotypic states through immune-parenchymal ligand-receptor interactions during psoriasis. (**Figure 5**).

To explore the mechanism, we found that chemokines CXCL1, CXCL2 and CXCL3 were decreased on siMED1 cells after the M5 stimulation both on the RNA and protein level. We also found the number of chemotactic neutrophils was significantly diminished when co-cultured with human keratinocytes in siMED1 group. These results proved that the diminished neutrophil accumulation on MED1 deficiency of keratinocytes dues to the decreased neutrophil chemotaxis. In order to further investigate how MED1 regulate the chemokines, dual-luciferase reporter results demonstrated that MED1 deficiency significantly attenuated luciferase activity of CXCL1, CXCL2 and CXCL3. Collectively, these results demonstrated that MED1 deficiency suppressed neutrophil chemotaxis by reducing its transcriptional binding to chemokines CXCL1/2/3 (**Figure 6**). We agree that beyond the direct transcriptional regulation of CXCLs, MED1 may engage additional signaling cascades to influence psoriasis pathogenesis. To systematically explore this, we performed KEGG pathway enrichment analysis on the differentially expressed genes derived from the entire cell populations in our scRNA-seq dataset (comparing WT versus cKO mice). Notably, the PI3K–Akt signaling pathway emerged as one of the most significantly enriched pathways, suggesting that MED1 deficiency may involve in PI3K-Akt signaling axis to affect psoriasis (**Supplementary Figure 4**). This finding will be explored in ongoing study.

Our study illustrated a mechanism of the epidermal Med1 deficiency inhibited the neutrophil accumulation, thus alleviating IMQ-induced psoriasis. We also identified three novel subtypes of neutrophils to give insights into the dynamic alterations of neutrophils in psoriasis at the single-cell level.

## Data Availability

The data presented in the study are deposited in the https://www.ncbi.nlm.nih.gov/sra/PRJNA1527074, accession number PRJNA1527074.

## Supplementary material

Supplementary Figure 1 The quality control of scRNA-seq.

Supplementary Figure 2 The pathways enriched in neutrophil Nc cluster. **(A)** and **(B)** The pathways enriched in neutrophil Nc cluster from WT and cKO groupswith GO **(A)** and KEGG **(B)** analysis.

Supplementary Figure 3 Pathways enriched in macrophage subpopulations from the WT and cKO groups from GO analysis.

Supplementary Figure 4 Pathways enriched of scRNA-seq from WT and cKO groups with KEGG analysis.
