# Supplementary material for: Keratinocyte-derived CXCL-CXCR2 ligand-receptor axis by MED1 orchestrates neutrophil phenotypes during psoriasis
Source: Front Immunol. 2026 Sep 30;17:1935554. doi: 10.3389/fimmu.2026.1935554 (PMC13661492; doi:10.3389/fimmu.2026.1935554)
Supplement: Supplementary file 1 [file SupplementaryFile1.pdf]

frontiers

The International Society for the Advancement of Cytometry (ISAC) has highlighted the importance of including comprehensive methodological information to ensure data reproducibility and reliability. In line with this, Frontiers in Immunology now requires authors to submit a checklist for manuscripts that involve flow or mass cytometry. This checklist helps standardize the reporting process, improving the quality and transparency of published data. By doing so, we support scientific progress, making it easier for other researchers to replicate and validate experiments.

This form should be submitted with any manuscripts using flow or mass cytometry.

Sample/specimen/material description

- [ ] Total blood
- [ ] PBMCs
- [x] Organ digests

Other_______________________________________

Did the samples suffer any treatment before or after incubation with the antibodies?

- [ ] Drug ___________________________________
- [ ] Cell permeabilization _______________________
- [ ] Dye _____________________________________
- [ ] Propidium iodine
- [x] Not applicable

Other _______________________________________

Instrument and antibodies

Name of the Cytometer BD LSR Fortessa

Antibodies and targets	Fluorochrome/ Metal	Catalog number/Company
e.g. anti-CD4	FITC	Cat. XXX/ XXX ltd.
anti-CD45	APC/Cy7	157203/Biolegend

anti-F4/80	FITC	101205/Biolegend
anti-Ly6G	Perp/Cy5.5	127615/Biolegend
anti-CD11c	PE	117307/Biolegend
anti-CD3e	Perp/Cy5.5	152321/Biolegend
anti-CD16/CD32		101301/Biolegend

Data analyses

1. Name of the software FlowJo 10.8.1 ____________________________________
2. Reference gating strategy in the manuscript or supplementary material

Gating strategy in (eg Figure X) Materials and Methods ______________________________
